# Supplementary material for: Oral Health Status, Knowledge, and Behaviours of People with Diabetes in Sydney, Australia
Source: Int J Environ Res Public Health. 2021 Mar 26;18(7):3464. doi: 10.3390/ijerph18073464 (PMC8037358; doi:10.3390/ijerph18073464)
Supplement: Supplementary file 1 [file ijerph-18-03464-s001.zip › ijerph-1127822-suppl. final/ijerph-1121822-suppl. final.pdf]

Table S1. Recoded Study Variables.

Variable Type	Study Variables	Original	Re-coded for Analysis
Demographic Variables	Country of Birth	Australia	Australia
		Other Countries	Overseas
	Language spoken at home	English	English
		Other languages	Other than English
Socio-economic Variables	Education Attained	No Schooling	No schooling
		Primary School	Primary School
		Secondary School	High School
		High School	
		TAFE	TAFE
		University	University
	Combined annual household income	Less than $40,000	Less than $40,000
		$40,000 to less than $60,000	$40,000 to less than $60,000
		$60,000 to less than $80,000	$60,000 to less than $80,000
		$80,000 to less than $100,000	$80,000 to less than $100,000
		$100,000 to less than $120,000	$100000 and more
		More than $120,000	
		Don't know	Missing (removed from analysis)
Health Specific Variables	Health Insurance Status	Yes	Have Health Insurance
		No	Do Not Have Health Insurance
		Don't know	Missing (removed from analysis)
	Knowledge about oral health	Item 1, 3, 8, 9 & 10-<br>True & Don't know<br>False	 Incorrect<br>Correct
		Item 2, 4, 5, 6, 6 & 7-<br>False & Don't' know<br>True	 Incorrect<br>Correct
	Oral Health Status	Excellent	Good to Excellent
		Very good	
		Good	
		Fair	Fair to poor
		Poor	

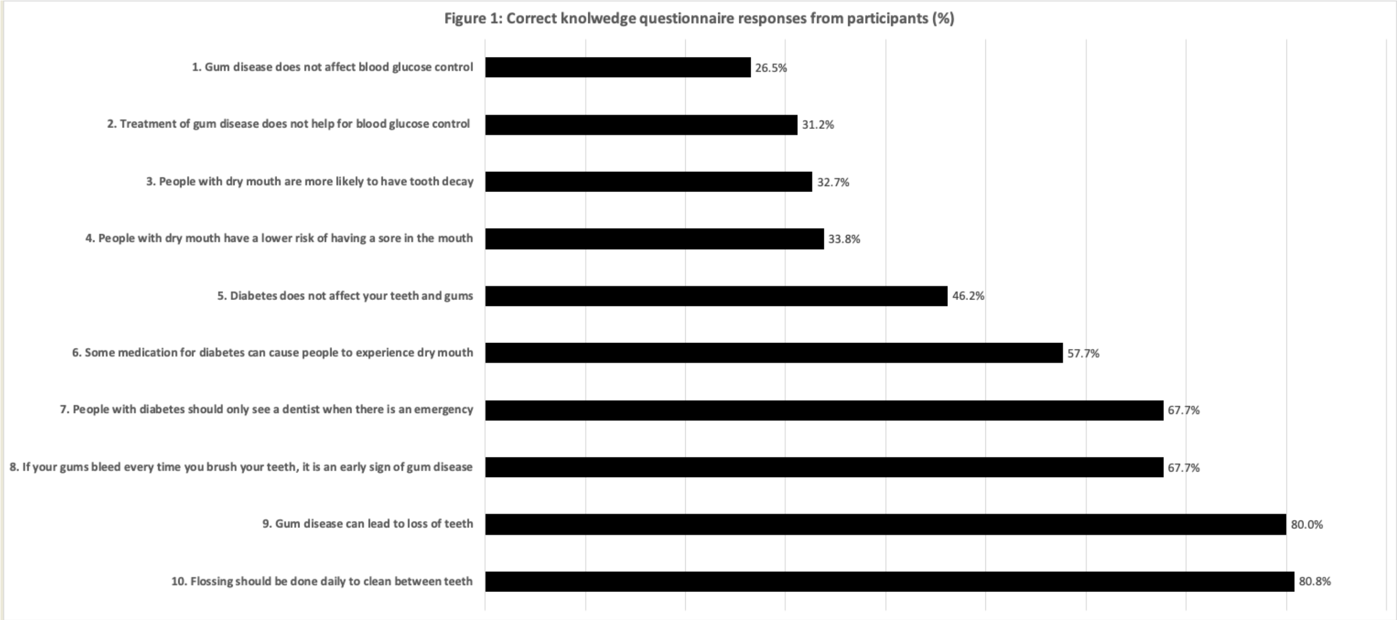

Figure S1. Correct knowledge questionnaire responses from participants.

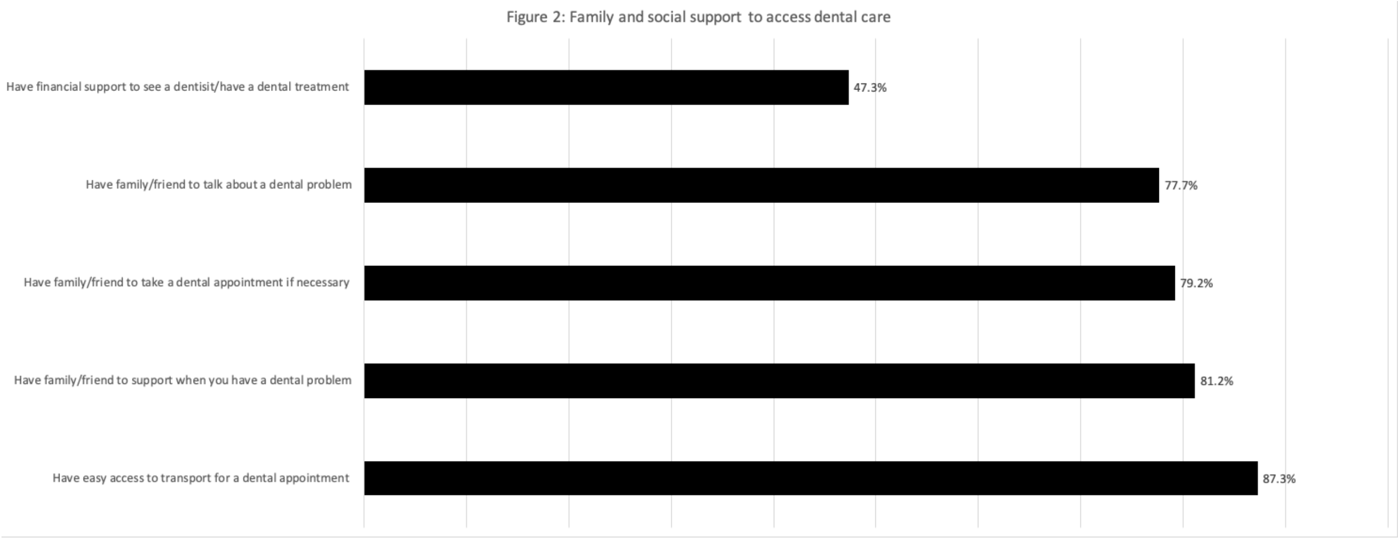

Figure S2. Family and social support to access dental care.
