# Supplementary material for: Oral Health Status, Knowledge, and Behaviours of People with Diabetes in Sydney, Australia
Source: Int J Environ Res Public Health. 2021 Mar 26;18(7):3464. doi: 10.3390/ijerph18073464 (PMC8037358; doi:10.3390/ijerph18073464)
Supplement: Supplementary file 1 [file ijerph-18-03464-s001.zip › ijerph-1127822-suppl. final/Supplementary File S1.pdf]

Appendix A S1 Survey

SURVEY QUESTIONNAIRE

DIABETES AND ORAL HEALTH (DIOH) RESEARCH

Please answer all questions.

1. DENTAL HEALTH STATUS

First, some questions about your dental health.

1. How would you describe the health of your teeth and mouth? *Mark ☑ one box*.

❒1 Excellent ❒2 Very Good ❒3 Good ❒4 Fair ❒5 Poor

2. Do you currently have any problems or concerns with your teeth, gums or mouth?

❒1 Yes ❒2 No

If yes, what are your main problems/concerns? (*Mark* ☑ all that apply)

Bleeding gums	❒1
Pain in your teeth and/or gums	❒2
Gaps between your teeth	❒3
Loose teeth	❒4
Dry mouth	❒5
Bad taste or smell in your mouth	❒6
Other problems ______________________	❒7

3. The following statements relate to issues you might have faced because of dental problems. Please indicate how often you have faced these issues. *Mark ☑ one box for each question.*

	Never	Hardly ever	Occasionally	Fairly often	Very often
Have you had trouble *pronouncing any words* because of problems with your teeth, mouth or dentures?	❒1	❒2	❒3	❒4	❒5
Have you felt that your *sense of taste* has worsened because of problems with your teeth, mouth or dentures?	❒1	❒2	❒3	❒4	❒5
Have you had a *painful aching* in your mouth?	❒1	❒2	❒3	❒4	❒5
Have you found it *uncomfortable to eat any foods* because of problems with your teeth, mouth or dentures?	❒1	❒2	❒3	❒4	❒5
Have you been *self-conscious* because of your teeth, mouth or dentures?	❒1	❒2	❒3	❒4	❒5
Have you *felt tense* because of problems with your teeth, mouth or dentures?	❒1	❒2	❒3	❒4	❒5

Has your *diet been unsatisfactory* because of problems with your teeth, mouth or dentures?	❒1	❒2	❒3	❒4	❒5
Have you had to *interrupt meals* because of problems with your teeth, mouth or dentures?	❒1	❒2	❒3	❒4	❒5
Have you found it *difficult to relax* because of problems with your teeth, mouth or dentures?	❒1	❒2	❒3	❒4	❒5
Have you been a bit *embarrassed* because of problems with your teeth, mouth or dentures?	❒1	❒2	❒3	❒4	❒5
Have you been a bit *irritable with other people* because of problems with your teeth, mouth or dentures?	❒1	❒2	❒3	❒4	❒5
Have you had *difficulty doing your usual jobs* because of problems with your teeth, mouth or dentures?	❒1	❒2	❒3	❒4	❒5
Have you felt that life in general was *less satisfying* because of problems with your teeth, mouth or dentures?	❒1	❒2	❒3	❒4	❒5
Have you been *totally unable to function* because of problems with your teeth, mouth or dentures?	❒1	❒2	❒3	❒4	❒5

2. KNOWLEDGE ABOUT DENTAL HEALTH

4. Please choose an answer for the following questions based on your current knowledge. *Mark ☑ one box for each statement.*

	True	False	Don't know
Diabetes does not affect your teeth and gums	❒1	❒2	❒3
Some medications for diabetes can cause people to experience dry mouth	❒1	❒2	❒3
People with dry mouth have a lower risk of having a sore in the mouth	❒1	❒2	❒3
People with dry mouth are more likely to have tooth decay	❒1	❒2	❒3
Flossing should be done daily to clean between teeth	❒1	❒2	❒3
If your gums bleed every time you brush your teeth, it is an early sign of gum disease.	❒1	❒2	❒3
Gum disease can lead to loss of teeth	❒1	❒2	❒3
Gum disease does not affect the blood glucose control	❒1	❒2	❒3
People with diabetes should only see a dentist when there is an emergency	❒1	❒2	❒3
Getting treatment of gum disease (deep cleaning of your tooth and root surface) does not help for blood glucose control	❒1	❒2	❒3

3. ATTITUDES TOWARD DIABETES AND DENTAL HEALTH

5. How would you describe the importance of the following activities? *For each statement, mark ☑ one box that comes closest to the way you think.*

	Very important	Fairly important	Important	Slightly important	Least important
Doing exercise regularly	❒1	❒2	❒3	❒4	❒5
Eating a healthy diet	❒1	❒2	❒3	❒4	❒5
Brushing teeth twice a day	❒1	❒2	❒3	❒4	❒5
Use of floss/interdental brush to clean between teeth at least once a week	❒1	❒2	❒3	❒4	❒5
Visit a dentist at least once a year	❒1	❒2	❒3	❒4	❒5
Visit a GP/diabetes specialist regularly	❒1	❒2	❒3	❒4	❒5
Keeping good control of your diabetes	❒1	❒2	❒3	❒4	❒5

6. These questions are about how you feel about your teeth and gum. *For each question, mark ☑ one box that comes closest to the way you feel.*

	Strongly agree	Agree	Disagree	Strongly disagree
Taking care of my teeth and gums is as important as taking care of my general health.	❒1	❒2	❒3	❒4
If someone tells me that I am at risk of teeth and gum problems, I will regularly see a dentist.	❒1	❒2	❒3	❒4

4. DENTAL CARE PRACTICES

The following questions are about your dental care behaviours.

7. Have you seen a dentist within the last 12 months? *Mark ☑ one box.*

❒1 Yes ❒2 No → ***go to Q9***

8. What was the main reason you last visited the dentist? *Mark ☑ all that apply.*

Check-up/exam/cleaning	❒1
Dental problems	❒2
Other ____________________	❒3
Don’t know	❒4

9. What was the main reason behind not visiting dentist? *Mark ☑ all that apply.*

Could not afford the cost	❒1
Did not want to spend the money	❒2
Insurance did not cover treatments	❒3
Dental office is too far away	❒4
Dental office is not open at convenient times	❒5
Afraid or do not like dentist	❒6
Unable to take off time from work	❒7
Too busy	❒8
I did not have any dental problems	❒9
I did not think anything serious was wrong/expected dental problems to go away	❒10
Other ______________________________	❒11
Don't know	❒12

10. In your last visit, did dentist tell you about benefits of: *Mark ☑ one box in each statement*.

a. Checking your blood sugar.	❒1 Yes	❒2 No	❒3 Don't know
b. Giving up cigarettes or other types of tobacco.	❒1 Yes	❒2 No	❒3 Don't know

11. How often do you brush your teeth? *Mark ☑ one box.*

More than twice a day	❒1
Twice a day	❒2
Once a day	❒3
A few times a week	❒4
Never	❒5

12. Which of the following do you use? *Mark ☑ all that apply.*

Fluoride toothpaste	❒1
Mouthwash	❒2
Sugar free chewing gum	❒3
None	❒4

13. In the last seven days, how many days did you use dental floss/interdental brush to clean between your teeth?

Enter number of days ☐

5. DIABETES CARE PRACTICES

14. During the past 12 months, how many times have you seen the following health care professional for your diabetes?

1 Diabetes Educators	Enter number of times	☐	None ❐
2 Diabetes Specialist	Enter number of times	☐	None ❐
3 General Practitioners (GPs)	Enter number of times	☐	None ❐
4 Dietitian/Nutritionist	Enter number of times	☐	None ❐
5 Other ______________________	Enter number of times	☐	None ❐

15. Have you ever received any information about teeth and gums from your diabetes care providers (such as, diabetes educators, GPs/specialist and dietitians)? *Mark ☑ one box.*

❐1 Yes ❐2 No → ***go to Q16***

a. What information/service did you receive? *Mark* ☑ all that apply.

1 Examination/looking of your teeth and gums	❐1 Yes	❐2 No
2 Brushing your teeth regularly	❐1 Yes	❐2 No
3 Clean between your teeth using floss or interdental brush regularly	❐1 Yes	❐2 No
4 Visit to a dentist or dental hygienist every year	❐1 Yes	❐2 No
5 Brochure/pamphlet about diabetes and dental health	❐1 Yes	❐2 No
6 Referral letter to a dentist	❐1 Yes	❐2 No

7 If other, please specify____________________________________

b. Who provided you information/service about teeth and gums? *Mark ☑ all that apply.*

Diabetes Educators	❐1
Diabetes Specialist	❐2
General Practitioners (GPs)	❐3
Dietitian/Nutritionist	❐4
Other________________________	❐5
Cannot recall	❐6

16. How would you think the following services provided by diabetes care providers (such as, diabetes educators, GPs/specialist and dietitians)? *Mark ☑ one box to each statement*.

	Yes	No	Don't know
Do you think diabetes care providers could help you to identify teeth and gum problems?	❒1	❒2	❒3
Would you consider advice related to teeth and gum given by diabetes care providers?	❒1	❒2	❒3
Do you think diabetes care providers have good knowledge about teeth and gums to advise you?	❒1	❒2	❒3
Would you make an appointment to see a dentist if you were given a dental referral by a diabetes care provider?	❒1	❒2	❒3

17. Which of the following health care professionals would be the most appropriate to provide you information about teeth and gums? *Mark ☑ one box.*

Diabetes Educator	❒1
GPs/diabetes Specialist	❒2
Dietitian	❒3
Don't know	❒4

18. How likely are you to participate in these services if they are provided by diabetes educators? *Mark ☑ one box to each statement*.

	Extremely likely	Very likely	Likely	Not very likely	Not likely at all
Diabetes educators providing you information about teeth and gums using leaflets or pamphlets	❒1	❒2	❒3	❒4	❒5
Diabetes educators providing you dental advice	❒1	❒2	❒3	❒4	❒5
Diabetes educators asking questions to find out about your teeth and gum problems	❒1	❒2	❒3	❒4	❒5
Diabetes educators looking your mouth and teeth	❒1	❒2	❒3	❒4	❒5
Diabetes educators providing you referrals to a dentist	❒1	❒2	❒3	❒4	❒5
Having priority access to public dental service	❒1	❒2	❒3	❒4	❒5
Receiving free vouchers to attend private dental clinics	❒1	❒2	❒3	❒4	❒5

6. FAMILY AND SOCIAL SUPPORT

19. Some *questions relate to your family/social support to access dental care. Mark ☑ one box to each question.*

	Yes	No
Do you have someone (family or friend) to give you support when you have a dental problem?	❒1	❒2
Do you have someone (family or friend) to talk about your dental problems if you have any?	❒1	❒2
Do you have someone (family or friend) to who can take you to dental appointments if necessary?	❒1	❒2
Do you have financial support to see a dentist or have dental treatment if necessary?	❒1	❒2
Do you have easy access to transport if you need to go to dental appointment?	❒1	❒2

1. What is your date of birth?

7. FINALLY, SOME QUESTIONS ABOUT YOU (DEMOGRAPHICS)

2. Gender ❒1 Male ❒2 Female

3. In which country were you born?________________________

4. What language do you speak at home? __________________

5. What is your post code? ______________________________

6. When were you first diagnosed with diabetes? Year ________or Age_________

7. Type of diabetes *Mark* ☑ *one box.*

❒1 Type1 ❒2Type2 ❒3 Gestational diabetes mellitus (GDM) ❒3 Other_________

❒4 Don’t know

8. Thinking about the most recent blood glucose level test, how was your result? *Mark* ☑ *one box.*

❒1Too high ❒2 About right ❒3 Too low ❒4 Don’t know

9. Are you a current smoker? ❒1 Yes ❒2 No

10. Do you drink alcohol? ❒1 Yes ❒2 No

11. Do you have any other chronic diseases, apart from diabetes? *Mark* ☑ *one box.*

❒1 Yes, please specify _________________________ ❒2 No

12. Are you currently working? *Mark ☑ one box.*

❒1 Working full time ❒2 Working part time ❒3 Not working

13. What is your highest educational qualification? *Mark ☑ one box.*

Primary school	❒1
Secondary school	❒2
High school	❒3
TAFE	❒4
University	❒5
No schooling	❒6

14. What is your marital status? *Mark ☑ one box.*

❒1 Single ❒2 Married/partnered ❒3 Divorced ❒4 Widowed

15. What is your combined annual household income? *Mark ☑ one box.*

Less than $40,000	❒1
$40,000 to less than $60,000	❒2
$60,000 to less than $80,000	❒3
$80,000 to less than $100,000	❒4
$100,000 to less than $120,000	❒5
More than $120,000	❒6
Don't know	❒7

16. Do you have private health insurance? *Mark ☑ one box.*

❒1 Yes ❒2 No ❒3 Don't know

17. Do you currently have the following cards? *Mark ☑ all that apply.*

Pensioner concession card,	❒1Yes	❒2 No	❒3 Don't know
Health care card	❒1Yes	❒2 No	❒3 Don't know
Department of Veterans Affairs card	❒1Yes	❒2 No	❒3 Don't know

THANK YOU FOR PARTICIPATING IN THIS SURVEY
